# Development of New Microsatellite DNA Markers from *Apostichopus japonicus* and Their Cross-Species Application in *Parastichopus parvimensis* and *Pathallus mollis*

**DOI:** 10.3390/ijms12095862

**Published:** 2011-09-09

**Authors:** Meijie Liao, Yingeng Wang, Xiaojun Rong, Zheng Zhang, Bin Li, Lan Wang, Guiping Chen

**Affiliations:** Qingdao Key Laboratory for Marine Fish Breeding and Biotechnology, Yellow Sea Fisheries Research Institute, Chinese Academy of Fishery Sciences, 106 Nanjing Road, Qingdao 266071, China; E-Mails: liaomj@ysfri.ac.cn (M.L.); rongxj@ysfri.ac.cn (X.R.); zhangzheng@ysfri.ac.cn (Z.Z.); libin841008@163.com (B.L.); wanglan0829@yahoo.cn (L.W.); chengp@ysfri.ac.cn (G.C.)

**Keywords:** sea cucumber, *Apostichopus japonicus*, Microsatellite DNA marker, cross-species amplification

## Abstract

Twenty microsatellite DNA markers were developed for sea cucumber and used to investigate polymorphisms of 60 wild *Apostichopus japonicus* individuals collected from China. It revealed that all the markers were polymorphic. A total of 164 alleles were detected at 20 loci. The number of alleles per locus varied from 3 to 17 with an average of 8.2, and the expected heterozygosities of each locus ranged from 0.03 to 0.89 with an average of 0.64. Cross-species amplification was also conducted in *Parastichopus parvimensis* collected from the United States and *Pathallus mollis* collected from Peru. The result showed that 17 loci amplified *Parastichopus parvimensis* DNAs while only 4 loci could amplify *Pathallus mollis* DNAs. All of the polymorphic markers would be useful for future genetic breeding and the assessment of genetic variation within sea cucumbers.

## 1. Introduction

Sea cucumber (*Apostichopus japonicus*) belongs to the phylum Echinodermata, class Holothuroidea. It is a common species distributed in shallow waters along northern Asian coasts in China, Japan, Russia and Korea [1,2]. It is the most valuable species in China due to its high nutritional and medicinal properties. However, resources of natural sea cucumber in Asian coast have decreased dramatically due to overfishing and severe environmental pollutions. Artificial propagation techniques for sea cucumber were developed in China in the 1980s. In recent years, sea cucumber aquaculture has developed rapidly, and the production reached 102,159 tons valued at 20 billion Yuan in 2009 [3]. However, due to inappropriate broodstock management, low-grade artificial seeds have caused tremendous decrease of genetic diversity and deterioration of disease resistance and growth performance. Thus, it is very important to carry out research on sea cucumber genetics, such as establishing broodstock for selection, estimating genetic parameters for growth and anti-bacteria, establishing genetic linkage map and development of molecular markers for species identification and population genetics studies.

To date, genetic marker systems including allozyme [4,5], AFLP [6], microsatellite [7–10], ISSR [11], mtDNA [12] and SNP [13] have been used in genetic studies. Of all the molecular marker types, microsatellite markers have been proven to be an extremely valuable tool for genetic studies and conservation and management of genetic resources. However, only 65 microsatellite markers developed from an enriched microsatellite library have been reported in *A. japonicus* until now [7,14]. Additional highly informative microsatellite markers are necessary to investigate the status of the wild resources and the genetic linkage map construction.

In the present study, we reported development of microsatellite in sea cucumber (*A. japonicus*) and characterization of the microsatellite markers by genotyping 60 individuals sampled from a wild population. Additionally, cross-species amplification was carried out to determine the potential for cross utility, and amplification of identified markers were assessed in two related species—warty sea cucumber (*Parastichopus parvimensis*) and black sea cucumber (*Pathallus mollis*).

## 2. Results and Discussion

Out of 252 random selected recombinant clones, 142 clones (56.3%) were found to contain inserts with a microsatellite motif in the middle position indicating that the enrichment was highly effective. Of the 142 clones surviving PCR screening, 118 (83.1%) contained a microsatellite motif in the middle position after being sequenced (Table 1). According to Weber’s (1990) classification rules [15], the sequences were divided into three categories: 83 perfect repeat sequences without interruptions in the runs of CA or GT di-nucleotides (70.4% of total), 30 imperfect repeat sequences with one or more interruptions in the run of repeats (25.4%), and 5 compound repeat sequences with adjacent tandem simple repeats of a different sequence (4.2%). When classified using the repeat sequence type, 116 clones (98.3%) had di-nucleotide repeats among which 88 clones’ repeat number was between 5 to 9 and 28 clones’ repeat number was higher than 10, the other two clones had tetra-nucleotide motifs.

As the first batch, twenty pairs of primers were designed according to 30 sequences and used to investigate polymorphism of 60 sea cucumber individuals. All of the tested 20 primer pairs showed clear band patterns and polymorphic (Table 2). A total of 164 alleles were detected at 20 loci and the total of effective allele numbers was 84. The number of alleles (*N*_a_) at each locus ranged from 3 (AJ08) to 17 (AJ07) with an average of 8.2. The effective allele number (*N*_e_) ranged from 1(AJ08) to 8.3 (AJ01) with an average of 4.2. The difference between Na and Ne was caused by the uneven frequency of each allele. As to the relationship between the number of microsatellite repeats and polymorphism, many scholars have different views. Qu suggested that the polymorphism would be higher when the number of microsatellite repeats increased [16]. Zheng considered that many high polymorphic loci would be missed if only loci with high number of microsatellite repeats were chosen in genetic research [17]. The result of this experiment showed that there is no relationship between the number of microsatellite repeats and polymorphism, which was consistent with Zheng.

The observed heterozygosities (*H*_o_) of each locus ranged from 0.02 (AJ10) to 0.79 (AJ09) with an average of 0.38, and the expected heterozygosities (*H*_e_) of each locus ranged from 0.03 (AJ08) to 0.89 (AJ01) with an average of 0.64. According to the polymorphic index content (PIC) value of each locus, three loci (AJ04, AJ08 and AJ09) were low polymorphic (PIC < 0.25), three loci were moderate polymorphic (0.25<PIC < 0.5), and the other 14 loci were high polymorphic (PIC > 0.5). None of the loci showed significant linkage disequilibrium. After sequential Bonferroni correcting for multiple tests, fourteen loci were found to depart significantly from Hardy-Weinberg equilibrium (HWE). Further tests indicated that heterozygote deficiency at these loci was responsible for the departure. Another possible explanation for the departure from Hardy-Weinberg equilibrium is the dramatic decline in spawning populations, and non-random mating or genetic bottlenecks.

Cross-species amplification test showed that only 4 loci were successfully cross-amplified in *P. mollis* (Table 2), while 17 loci (85%) could get cross-amplification in *P. parvimensis*. It confirmed that microsatellite markers developed in *A. japonicus* could be used for related sea cucumber species.

## 3. Experimental Section

### 3.1. DNA Extraction

Sixty individuals of sea cucumber (*A. japonicus*) were collected from Qingdao coast in China. Warty sea cucumber (*Parastichopus parvimensis*) collected from USA and black sea cucumber (*Pathallus mollis*) collected from Peru in dried hard form were bought from the supermarket. The dried sea cucumbers were immersed in pure water in order to get water raise sea cucumber. Genomic DNA was extracted from longitudinal muscle using Mollusc DNA kit (Omega, USA). The extracted genomic DNA was stored at −20 °C until genotyping.

### 3.2.Microsatellite-Enriched Library Construction

Microsatellite-enriched library was conducted using the FIASCO (Fast Isolation by AFLP of Sequences Containing Repeats) method described in detail by Zane *et al*. in 2002 [18] with minor modification. Genomic DNA was digested with *Mse* I at 37 °C for 3 h and ligated with a synthesized *Mse* I adaptor (5′-TAC TCA GGA CTC AT-3′/5′-GAC GAT GAG TCC TGA G-3′) using T4 DNA ligase (Sangon, Shanghai, China). The digestion-ligation mixture was amplified using the adaptor-specific primer (5′-GAT GAG TCC TGA GTA A-3′). Microsatellite-containing fragments were selectively enriched, captured and washed using biotinylated (CA)_12_ and Streptavidin Magne Sphere^®^ Paramagnetic Particles (Promega, USA). Fragments containing microsatellites were ligated with the pMD18-T (TaKaRa, Dalian, China) vector and transferred into *E. coli* competent cell JM109 (TaKaRa, Dalian, China) by electroporation.

### 3.3. Isolation of Microsatellite-Containing DNA Fragments and Primer Design

In order to check whether the microsatellite motif located in the middle of the insert, each recombinant was subjected to three individual PCR screening using two universal sequencing primer and (CA)_12_DN oligonucleotide. In the first reaction, universal forward and universal reverse sequencing primer were used; in the second reaction, universal forward sequencing primer and (CA)_12_DN oligonucleotide were used; in the third reaction, universal reverse sequencing primer and (CA)_12_DN oligonucleotide were used. Recombinant clones that produced products in obviously different length between the first reaction and the second or the third reaction were sequenced and trimmed. The sequencing data were scanned using the software SSRHunter V1.3 [19]. Sequences with microsatellite motifs and flanking regions were selected for PCR primer design with the help of Primer Premier [20].

### 3.4. PCR Amplification and Genotyping

Microsatellite primers designed were used to amplify genomic DNA of 60 sea cucumber individuals, 5 warty sea cucumber individuals and 5 black sea cucumber individuals. The PCR mixture contained 1× buffer, 1.5 mmol/L MgCl_2_, 200 μmol/L dNTP (each), 200 μmol/L primers (each direction) and about 50 ng genomic DNA. The PCR conditions were denaturing at 94 °C for 1 min, followed by 30 cycles of 1 min at 94 °C, 1 min at annealing temperature, and 1 min at 72 °C, with a final extension for 5 min at 72 °C. The optimized annealing temperatures of different primer pairs were listed in Tm column of Table 2. The PCR product was separated on a 6% denaturing polyacrylamide gel and visualized by silver staining. Allele size was determined with software Quantity One V4.62 (Bio-rad) by referring to 20bp DNA ladder marker (TaKaRa, Dalian, China).

### 3.5. Genetic Data Analysis

Popgene version 1.32 [21] was used to calculate the number of alleles (Na), the number of effective alleles (Ne), observed heterozygosity (Ho) and expected heterozygosity (He). Polymorphism information content (PIC) of each locus was calculated according to Botsein (1980) [22]. Hardy-Weinberg equilibrium and linkage disequilibrium test were conducted using Genepoponline version [23]. Significance criteria of all multiple tests were corrected following sequential Bonferroni correcting [24].

## 4. Conclusions

In the present study, microsatellite-enriched genomic library of sea cucumber (*Apostichopus japonicus*) was constructed and a total of 20 novel genomic microsatellite DNA markers were developed. Most of the markers could amplify successfully in warty sea cucumber and black sea cucumber. The use of these microsatellite markers will certainly facilitate the management and exploration of the genetic resources of Holothuroidea and assist in their genetic improvement to some extent.

## Figures and Tables

**Table 1:** Classification of microsatellite DNA sequences obtained in this study.

Criterion	Category	No.of sequences	Percentage (%)
Weber [15]	Perfect	83	70.4
	Imperfect	30	25.4
	Compound	5	4.2

Repeat motif	Two bases 5 ≤ *n* ≤ 9	88	74.6
	Two bases *n*≥10	28	23.7
	Four bases	2	1.7

**Table 2:** Characterization of microsatellite DNA markers developed for sea cucumber (*Apostichopus japonicus*). *T*_m_: annealing temperature (°C); *N*_a_: allele number; *N*_e_: effective allele number, *H*_o_: observed heterozygosity, *H*_e_: expected heterozygosity, PIC: polymorphism index content, p: p value for exact test for Hardy-Weinberg equilibrium (HWE)

locus	Accession No.	Repeat motif	Primer sequence (5′→3′)	Size range (bp)	*T*_m_ (°C)	*N*_a_	*N*_e_	*H*_o_	*H*_e_	PIC	*p*	*Parastichopus parvi-mensis*	*Pathallus mollis*
**AJ01**	JF289179	(CA)_5_	F: TACGTCGTGAATGAATGT R: TCAGTTTTGAGACCCTTA	261–298	50	12	8.3	0.29	0.89	0.87	0.0000*	+	−
**AJ02**	JF289180	(AC)_5_	F: GGTTTTCTGTTGAGGCTGTGTGGAT R: AGTCCAAAGTTTTCTCCTGGGGTGA	209–226	62	8	5.4	0.64	0.82	0.79	0.0000*	+	−
**AJ03**	JF289181	(AC)_6_	F: TTCACAACGCATTCCAGT R: CATGTCATAAAGGCCAAA	132–168	52	11	5.9	0.73	0.84	0.81	0.2551	+	−
**AJ04**	JF289182	(AC)_3_AT(AC)_6_T(CA)_3_	F: TGTCCTGGCAAGAGAAAG R: GCATAGCCGTTACTTACC	178–223	54	6	1.2	0.15	0.16	0.16	0.0277	+	−
**AJ05**	JF289183	(AC)_3_AG(AC)_6_	F: CGATAACCCACTTGCTGC R: CGTGTTGTCCACTTCCAT	279–325	58	11	4.1	0.57	0.77	0.74	0.0000*	−	−
**AJ06**	JF289184	((AC)_6_	F: GTATCCACTACCCGTTTG R: AATTTCCTCGCATATCAC	210–246	52	5	1.4	0.27	0.28	0.26	0.0616	+	−
**AJ07**	JF289185	(GT)_5_ATGTAT(GT)_13_	F: GCGGGAATCTAAGGGATA R: GTGGGCACCAGAAACAAT	252–286	54	17	7.9	0.67	0.88	0.86	0.0000*	+	+
**AJ08**	JF289186	(CA)_7_TG(CA)_5_	F: ACCAAATATGAAAGCCAAGT R: CACGATGTCTGTTATGTAGCG	292–300	52	3	1	0.03	0.03	0.03	1.0000	+	−
**AJ09**	JF289187	(CA)_4_GACAG(AC)_6_	F: ACAAGCACGCAGGGTCAC R: CAGGGGAGGGGTTTCAGA	184–210	60	11	4.4	0.79	0.78	0.74	0.1605	+	−
**AJ10**	JF289188	(CA)_7_	F: TCCTTTACAAGCCGTTAT R: TTGTTTGAGGTTCGGGAT	188–205	50	4	2.1	0.02	0.53	0.44	0.0000*	+	−
**AJ11**	JF289189	C_11_(CA)_7_	F: TTTTCCGTACCATGACCG R: CCTAACCAAATAGAGCCACA	230–256	54	8	5.9	0.3	0.84	0.81	0.0000*	+	−
**AJ12**	JF289190	(CA)_8_	F: AGCACGAATCTTTCACCT R: CAATGGAAAATACAATGGG	163–189	50	7	3.1	0.48	0.68	0.64	0.0000*	+	+
**AJ13**	JF289191	(AC)_6_	F: GCTGCCTTTTAATTTCTG R: CAATGTGGTGGCTTCCTC	126–179	50	12	5.7	0.59	0.83	0.8	0.0000*	+	−
**AJ14**	JF289192	(AC)_8_	F: TTTCGAGGACGTGGTGAT R: GTCCCTCAGGTCTGTTATTG	172–199	56	8	4.5	0.26	0.78	0.75	0.0000*	+	+
**AJ15**	JF289193	(AC)_10_	F: ACCGTACCAAACCTCTCTT R: CCTTCTTACTAATACATCCCAG	153–186	53	9	7.3	0.22	0.87	0.85	0.0000*	+	−
**AJ16**	JF289194	(CA)_3_CG(CA)_5_	F: GCCACTATTCATGTCTTCG R: GCAACCATTTACAACCCT	282–325	52	9	4.4	0.48	0.78	0.74	0.0000*	+	−
**AJ17**	JF289195	(AC)_5_GC(AC)_4_	F: ATCCAACTTGCCATTCTTC R: CTTTTTGATTCCTTGCCTG	116–156	53	7	2.1	0.52	0.53	0.48	0.0673	+	−
**AJ18**	JF289196	(CA)_5_	F: CCAGATTTCTCTAGTCCTTTTG R: GTATGCGGATGGGTTTCA	168–180	52	4	3.3	0.16	0.7	0.64	0.0000*	−	+
**AJ19**	JF289197	(CA)_5_…(CA)_5_	F: TTATGTTCCTAGAGCCTGT R: TGGGATGTACCCTAGAGT	257–288	51	4	1.1	0.02	0.08	0.08	0.0001*	−	−
**AJ20**	JF289198	C_12_(CA)_6_	F: TCCGAAAAAAGGTATTTGCTG R: ACTGGGCGAGATGATTGGT	311–325	55	8	4.8	0.38	0.8	0.76	0.0000*	+	−

*: departure from HWE after Bonferroni correction; +: success in cross-species amplification; −: unsuccess in cross-species amplification.
